# Synaptic Conversion of Chloride-Dependent Synapses in Spinal Nociceptive Circuits: Roles in Neuropathic Pain

**DOI:** 10.1155/2011/738645

**Published:** 2011-06-15

**Authors:** Mark S. Cooper, Adam S. Przebinda

**Affiliations:** Department of Biology, University of Washington, Seattle, WA 98195-1800, USA

## Abstract

Electrophysiological conversion of chloride-dependent synapses from inhibitory to excitatory function, as a result of aberrant neuronal chloride homeostasis, is a known mechanism for the genesis of neuropathic pain. This paper examines theoretically how this type of synaptic conversion can disrupt circuit logic in spinal nociceptive circuits. First, a mathematical scaling factor is developed to represent local aberration in chloride electrochemical driving potential. Using this mathematical scaling factor, electrophysiological symbols are developed to represent the magnitude of synaptic conversion within nociceptive circuits. When inserted into a nociceptive circuit diagram, these symbols assist in understanding the generation of neuropathic pain associated with the collapse of transmembrane chloride gradients. A more generalized scaling factor is also derived to represent the interplay of chloride and bicarbonate driving potentials on the function of GABAergic and glycinergic synapses. These mathematical and symbolic representations of synaptic conversion help illustrate the critical role that anion driving potentials play in the transduction of pain. Using these representations, we discuss ramifications of glial-mediated synaptic conversion in the genesis, and treatment, of neuropathic pain.

## 1. Introduction

**Figure 1:**
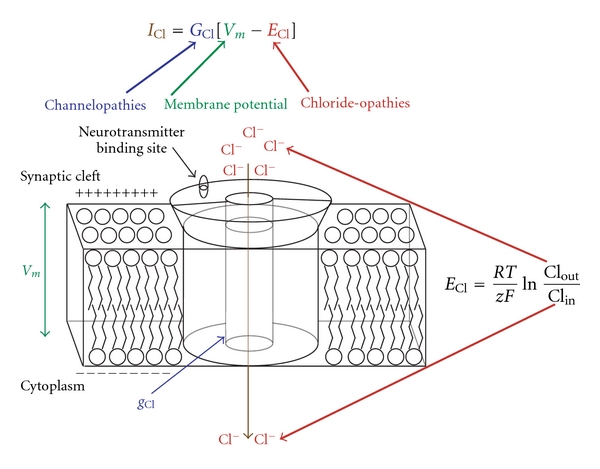
Ohm's law for a chloride current in a postsynaptic membrane. Total synaptic current for chloride, *I*_Cl_, is the summation of chloride currents flowing through individual chloride channels. The total driving potential Δ for chloride (see (1)) is given by [*V*_*m*_ − *E*_Cl_]. Chloride channelopathies (i.e., mutation of chloride channels) alter the ionic conductance of individual chloride channels, *g*_Cl_, thus producing pathological chloride currents. Pathological changes in the Nernst potential for chloride (i.e., chloride-opathies) can also produce aberrant chloride currents. Aberrations in *E*_Cl_ can result from pathological changes in either intracellular or extracellular chloride concentration.

**Figure 2:**
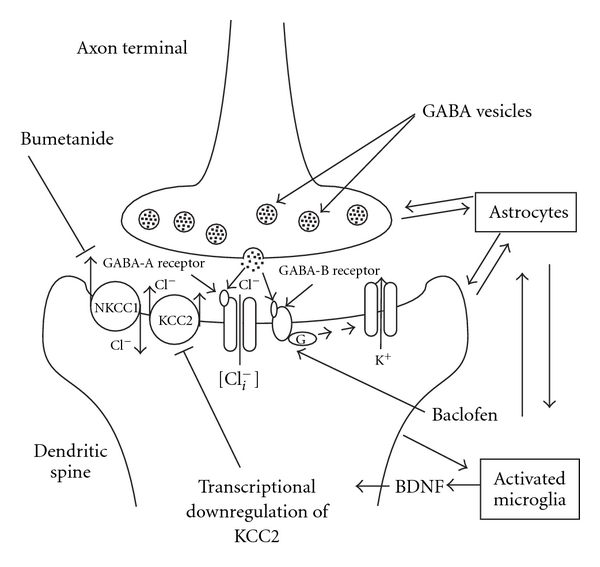
A “tetrapartite” synapse in the CNS: A GABAergic synapse modulated by reciprocal signaling and homeostatic interactions with neighboring astrocytes and microglia [7]. GABAergic synapses employ ionotropic (GABA-A receptors) and metabotropic (GABA-B) receptors to evoke postsynaptic potentials [1]. The ionotropic GABA-A receptor is a ligand-gated chloride channel. The metabotropic GABA-B receptor activates a hyperpolarizing potassium conductance through a G-protein-mediated pathway [1]. As part of their neuroprotective response, activated microglia release brain-derived neurotropic factor (BDNF) in response to neuronal or vascular injury. BDNF triggers a transcriptional downregulation of KCC2 (potassium-chloride cotransporter 2), leading to reduced chloride extrusion from the cell [3]. Pathological accumulation of intracellular chloride can be reduced by blocking the inward sodium-potassium-chloride cotransporter (NKCC1) with bumetanide [6]. Diminished functioning of the GABA-A receptor, because of a collapsed chloride gradient, could be compensated for applying baclofen, a GABA-B agonist [22]. As pain therapeutics, bumetanide and baclofen, offer different physiological approaches to deal with the chloride-opathy in the postsynaptic neuronal cell.

Neuropathic pain involves multiple interlocking molecular and cellular pathologies [1, 2]. One of these pathologies is the alteration of transmembrane chloride gradients in nociceptive neurons, induced by chemical signals released by neighboring activated glia [3–5]. It is now well established that aberrant transmembrane chloride gradients in nociceptive neurons can result in a pathological functioning of antinociceptive chloride-dependent synapses [6] (Figure 1). The transmembrane chloride gradient within a postsynaptic nociceptive neuron is therefore a key variable that determines the excitability of *tetrapartite synapses*, that is, neuronal synapses that are modulated by the actions of gliotransmitters released by neighboring astrocytes and microglia [7] (Figure 2). 

 Chloride-dependent synapses (e.g., GABAergic and glycinergic) play critical roles in the production of inhibitory tone in both the central (CNS) and peripheral mammalian nervous system (PNS) [6, 8]. As primary nociceptive afferents become inflamed, the sodium-potassium-chloride cotransporter, NKCC1, is thought to be upregulated [9]. It has been proposed that NKCC1 upregulation leads to excessive chloride accumulating in the terminals of primary afferents, which in turn leads to excessive presynaptic afferent depolarization (PAD) at these locations [9, 10]. Instead of producing inhibition at the terminals of primary nociceptive afferents, action potentials may be initiated by the excessive PAD, resulting in antidromic firing, a neuronal behavior known as a dorsal root reflex [8–11]. 

Dorsal root reflexes can cause CGRP (calcitonin gene-related peptide) and substance P to be released from C fibers in peripheral tissues. This pathological process is considered to be a potent mechanism for the generation of visceral hypersensitivity and dermal hyperalgesia [8–12]. In addition to antidromic firing, sites of excessive PAD in primary afferents can also ignite volleys of actions potentials into the dorsal horn, contributing to the generation of neuropathic pain [10, 11].

 Excessive PAD at the terminals of primary nociceptive afferents is not the only mechanism by which aberrant chloride homeostasis within nociceptive circuits can generate neuropathic pain. A separate mechanism involves a loss of chloride-dependent inhibitory tone within the central nervous system (CNS). This occurs when intracellular chloride concentration within nociceptive projection neurons becomes elevated above normal homeostatic levels [6]. 

**Figure 3:**
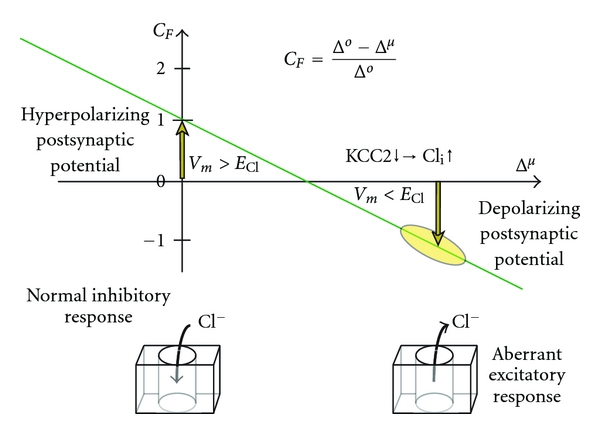
Electrophysiological conversion of chloride-dependent inhibitory synapses in the CNS. Neuropathologies arising from aberrant chloride homeostasis (i.e., chloride-opathies) can be mapped onto a graph of the Conversion Factor, *C*_*F*_, versus Δ^*μ*^. *C*_*F*_ is a scaling factor for synaptic response. Aberrant chloride current is scaled to the synaptic chloride current under normal conditions (i.e., Δ^*μ*^ = 0). The graph illustrates the special case of (8) where the resting membrane potential is unchanged in the cell with altered chloride driving potential (i.e., Δ^*V*^ = 0). Downregulation of Potassium Chloride Cotransporter (KCC2) leads to an increase in intracellular chloride. Hyperpolarizing GABAergic and glycinergic synapses become depolarizing synapses when *E*_Cl_* becomes more positive than *V*_*m*_.

 In rodents, terminals of injured peripheral neurons are known to release ATP and cytokines (e.g., monocyte chemotactic protein 1 (MCP-1) and fractalkine) that activate microglia within the spinal cord [13]. As part of their neuroprotective response, activated spinal microglia can release brain-derived neurotrophic factor (BDNF) [3]. BDNF produces a downregulation of the outward chloride cotransporter, KCC2, resulting in an increase of intracellular chloride in CNS neurons [3, 14]. When the transmembrane Nernst potential for chloride *E*_Cl_ becomes more positive than the resting potential *V*_*m*_ for the neuron (i.e., when *E*_Cl_ > *V*_*m*_), the electrochemical driving potential for chloride current becomes inverted. As a result, chloride-dependent inhibitory synapses undergo an electrophysiological conversion to become excitatory synapses (Figure 3). This synaptic behavior is known to occur in lamina I nociceptive projection neurons, contributing to tactile allodynia (amplified pain in response to a nonnoxious stimulus) [3]. 

 For nearly 20 years, the term *channelopathy* has been used to describe neurological and muscular pathologies arising from the molecular mutation of ion channels [15, 16]. A number of inherited neurological disorders, such as epilepsy, ataxia, and sensory pathologies have been linked to the altered ionic conductances of mutated ion channels [17, 18]. Channelopathies produce aberrant transmembrane ion currents, which consequently induce pathological firing patterns in affected neural circuits. 

 Pathologies in transmembrane ion currents can also occur when electrochemical driving potentials for specific ions become shifted outside of their normal range of function. A number of physiological stressors (e.g., ischemia, cellular injury, temperature shifts, toxins, pharmacological agents, endocrine signals), as well as tissue inflammatory processes, can produce pathological changes in transmembrane ion gradients and neuronal excitability. Transmembrane chloride gradients, in particular, play a key role in the genesis of inhibitory tone in the mature vertebrate nervous system [19, 20]. For this reason, aberrant transmembrane chloride gradients in postsynaptic neurons can potentially produce pathological effects in each function of the nervous system, including the sense of nociception [19]. 

 In this paper, we utilize the term *chloride-opathy* to describe neuropathologies that arise from aberrant chloride homeostasis. In the following section, we develop a mathematical scaling factor to quantify the effects of chloride-opathies on the function of chloride-dependent GABAergic and glycinergic synapses. We then use this scaling factor to portray how spinal nociceptive circuits become perturbed by chloride-opathies.

## 2. Methods and Modeling

### 2.1. Electrodynamics of Chloride-Dependent Synapses

 To model the altered behavior of a synapse with a chloride-opathy, it is particularly useful to reference the magnitude and sign of the altered chloride current to the normal chloride current. To accomplish this, one must first identify altered components to the chloride driving potential, and reference these changes to the norm-averaged components of the chloride driving potential.

 Total chloride current *I*_Cl_ through a synaptic membrane is given by:

(1) $$\overset{}{\underset{}{\sum }}i_{\text{Cl}}=I_{\text{Cl}}=G_{\text{Cl}}\left[V_{m}-E_{\text{Cl}}\right]=G_{\text{Cl}}\Delta ,$$

where *i*_Cl_ is the unitary current of a single chloride channel, and *G*_Cl_ is the total chloride conductance of the synapse [21]. The quantity [*V*_*m*_ − *E*_Cl_] is the difference between the resting membrane potential and the Nernst potential for chloride. In this paper, we hereafter refer to this difference in total electrochemical driving potential for chloride as the *chloride driving potential*. This total chloride driving potential is represented by the symbol, Δ. 

 Inward chloride flux across the cell membrane corresponds to an inhibitory, hyperpolarizing current. As intracellular chloride is increased in the postsynaptic cell, *E*_Cl_ > *V*_*m*_, the driving potential becomes inverted. The synaptic response becomes converted to an excitatory response. Hereafter, this process will be referred to as *synaptic conversion* (Figure 3).

 Synaptic conversion is directly tied to the transmembrane driving potential for the chloride ion. In this section, independent changes in the contributions of chemical potential and electrical potential to the total driving potential for chloride are first represented mathematically. Once such changes in chloride-dependent tone have been quantified, these changes can be placed within the context of the synaptic tone of spinal nociceptive circuits.

 To avoid the complexities of solving time-dependent differential equations, synaptic currents are often represented as steady-state conditions [21]. Synaptic currents are, therefore, often discussed using a steady-state version of Ohm's law. For a chloride-dependent synapse, with a given chloride conductance *G*_Cl_, membrane potential (*V*_*m*_) and the chloride Nernst potential (*E*_Cl_) are the two variables that will alter a chloride current from its normal value. These relationships can be seen in the following steady-state Ohmic relation:

(2) $$I_{\text{Cl}}^{*}=G_{\text{Cl}}\left[\left(V_{\text{rest}}-E_{\text{Cl}}^{o}\right)+\left(V_{m}-V_{\text{rest}}\right)-\left(E_{\text{Cl}}^{*}-E_{\text{Cl}}^{o}\right)\right].$$

Equation (2) can be rewritten to emphasize changes in the chloride driving potential:

(3) $$\begin{array}{c} I_{\text{Cl}}^{*}=G_{\text{Cl}}\left[\Delta^{o}+\Delta^{V}-\Delta^{\mu }\right]=G_{\text{Cl}}\Delta_{\text{Cl}}^{*} \end{array},$$

where *G*_Cl_ is the chloride conductance, and Δ_Cl_* is the altered electrochemical driving potential for chloride flux across the cell membrane, and

(4) $$\begin{array}{cc} \Delta^{o} & =V_{\text{rest}}^{o}-E_{\text{Cl}}^{o}\\ & =\text{net}\;\text{chloride}\;\text{driving}\;\text{potential}\;\\ & \;\text{at}\;\text{normal}\;\text{conditions}\;\left(\text{denoted}\;\text{by}\;o\right) \end{array}$$

(5) $$\begin{array}{cc} \Delta^{V} & =V_{m}-V_{\text{rest}}\\ & =\text{difference}\;\text{in}\;\text{membrane}\;\text{potential}\;\\ & \;\text{and}\;\text{resting}\;\text{membrane}\;\text{potential},\\ & =\text{perturbation}\;\text{in}\;\text{membrane}\;\text{potential} \end{array}$$

(6) $$\begin{array}{cc} \Delta^{\mu } & =E_{\text{Cl}}^{*}-E_{\text{Cl}}^{o}\\ & =\text{difference}\;\text{in}\;\text{chloride}\;\text{Nernst}\;\text{potential}\;\text{between}\\ & \;\text{perturbed}\;\left(\text{denoted}\;\text{by}\;\;*\right)\;\text{and}\;\text{normal}\;\text{cellular}\;\text{states}\\ & =\text{perturbation}\;\text{in}\;\text{chloride}\;\text{chemical}\;\text{potential}. \end{array}$$

*Note*. The symbol ∇ was used by Coull et al. [3] to denote the shift in the reversal potential for anion currents in lamina I projection neurons. In this paper, the term Δ^*μ*^ is used to emphasize deviations in the chloride chemical potential (denoted by *μ*) from resting conditions.

 This theoretical approach changes the representation of postsynaptic chloride current from being a function of membrane potential and chloride Nernst potential, that is, *I*_Cl_(*V*_*m*_, *E*_Cl_), into a function that is dependent on *deviations* from a normal resting chloride driving potential, Δ^*o*^. The postsynaptic chloride current, *I*_Cl_(Δ^*V*^, Δ^*μ*^, Δ^*o*^), becomes a function of the change in membrane potential, as well as the change in chloride chemical potential, from the normal resting chloride driving potential.

 There are two major advantages of representing *I*_Cl_ in this manner. Firstly, changes in the chloride driving potential are explicitly separated into two defined variables: changes in membrane potential (Δ^*V*^) versus changes in chloride chemical potential (Δ^*μ*^). Secondly, altered response in an aberrant chloride-dependent synapse can be quantitatively compared to the “normal” chloride driving potential, Δ^*o*^. Separating changes in membrane potential, Δ^*V*^, from changes in chloride chemical potential, Δ^*μ*^, provides an analytical framework to understand the process of synaptic conversion.

 In quantifying the magnitude of synaptic conversion, it is important to emphasize that the unitary conductance, *g*_Cl_, of an individual chloride ion channel is not altered between the two cellular states (i.e., normal versus chloride-opathy). An altered chloride driving potential does not change the transmembrane potential across a cell membrane. For this reason, the chloride channel will not change its three-dimensional structure, or its conductance, in response to a change in the transmembrane chloride gradient. Thus, the magnitude of individual chloride channel conductance, which remains unchanged, can be represented by

(7) $$g_{\text{Cl}}^{o}=g_{\text{Cl}}^{*}.$$

With no change in ionic conductance, aberrations in chloride current are linked solely to changes in chloride driving potential, Δ. One can directly compare aberrant transmembrane chloride current, *I*_Cl_*, to normal chloride current, *I*_Cl_, in a given chloride-dependent synapse. To accomplish this, it is useful to define a scaling factor, hereafter referred to as the synaptic *Conversion Factor*:

(8) $$C_{F}=\frac{I_{\text{Cl}}^{*}}{I_{\text{Cl}}^{o}}=\frac{\Delta^{o}+\Delta^{V}-\Delta^{\mu }}{\Delta^{o}}=\frac{\Delta^{*}}{\Delta^{o}}.$$

*C*_*F*_ is a unit-less variable, which references an aberrant chloride driving potential to the chloride driving potential in the normal state. The Conversion Factor can be viewed as a scaling factor for synaptic response. *C*_*F*_ can be used in combination with other unit-less variables, such as the electrotonic length *L* = *l*/*λ*, or electrotonic time *T* = *t*/*τ*, which are each scaled to inherent physical properties of the neuronal membrane (i.e., *l*: length of neuronal cable; *λ*: electrotonic length constant; *t*: real time; *τ*: electrotonic time constant of neuronal membrane).

In the case of a steady-state postsynaptic current,

(9) $$I_{\text{Cl}}^{*}=\left[\frac{\Delta^{o}+\Delta^{V}-\Delta^{\mu }}{\Delta^{o}}\right]\cdot I_{\text{Cl}}^{o}=C_{F}\cdot I_{\text{Cl}}^{o}.$$

Although chloride-opathies change the magnitude and/or direction of chloride-dependent synaptic currents, the passive electrotonic cable properties of affected neurons would remain unaltered. For example, if this aberrant postsynaptic current occurred at the terminus of a neuronal dendrite, the aberrant postsynaptic potential *V*_psp_* occurring at the neuronal soma would be approximated by:

(10) $$V_{\text{psp}}^{*}=\left(C_{F}I_{\text{Cl}}^{o}\right)\left(\frac{r_{m}}{A_{N}}\right)\left(\frac{L_{N}}{\tanh L_{N}}\right)=I_{\text{Cl}}^{*}\cdot R_{N},$$

where *r*_*m*_ is the specific resistance of the neuronal cell membrane, *A*_*N*_ is the area of neuron, *L*_*N*_ is the combined electrotonic length of the dendrite and soma, and *R*_*N*_ is the input resistance of the neuron (modified from (5.16) in [23]). Equation (10) illustrates that electrotonic properties of the neuronal membrane outside of the synapse remained unchanged by the change in chloride driving potential at the chloride-dependent synapse. Note that the neuronal input resistance, *R*_*N*_, is the same whether current in the chloride-dependent synapse is driven by a normal driving potential, Δ^*o*^, or by an altered driving potential, Δ*. 

 In the following section of this paper, we develop a set of symbols to represent the effects of chloride-opathies on nociceptive circuits. These symbols allow logic representations for synaptic response to be illustrated within the context of spinal nociceptive circuits.

### 2.2. Symbolic Representations of Synaptic Conversion

 The reversal potential, *E*_rev_, for a given ion channel is a fundamental concept in electrophysiology. Ionic current through the ion channel reverses direction when the driving potential (i.e., *V*_*m*_ − *E*_rev_) passes through the reversal potential for the ionic channel (Figure 3). A sustained shift in synaptic driving potential can thus reverse current flow through the ion channels of a synapse, and thus fundamentally change the functional response of the synapse (i.e., the magnitude and/or sign of the postsynaptic current). Previous authors have described this change as an inversion of the synaptic response [3, 5, 24]. Here, we employ the general term *synaptic conversion* to describe a continuum of aberrant synaptic response, which arises when the resting driving potential for the chloride ion deviates from time-averaged normalcy. 

 Changes in membrane potential are often coincident with the opening of a chloride-dependent synapse (3). In this case, *I*_Cl_ is determined by the local chloride driving potential, which is a combination of the local membrane potential and the local chloride chemical potential.

 In the following discussion, we deal with the special case of Δ^*V*^ = 0, to focus analysis on effects of chloride-opathies on the function of spinal reflex circuits. In this condition, changes in intracellular chloride concentration affect the Nernst potential for chloride, *E*_Cl_, but do not directly change the resting membrane potential of the neuron, *V*_*m*_. Thus, Δ^*μ*^ is the independent variable in the ionic transport equation that describes aberrant chloride current. This allows the altered response of chloride-dependent synapses to be viewed as being altered by changes in Δ^*μ*^, that is, the mathematical term that quantifies the magnitude of the chloride-opathy in the affected synapse. The term *synaptic conversion* is appropriate to use when the chloride-dependent synapse loses inhibitory strength and becomes progressively changed to an excitatory response as the chloride-opathy, Δ^*μ*^, increases in magnitude.

 To represent the concept of synaptic conversion in neuronal circuit diagrams, it is necessary to develop specific symbols that can be used to quickly identify an aberrant chloride-dependent synapse. These symbols can be incorporated into neuronal circuit diagrams to illustrate functional changes in synaptic behavior, as well as to visualize neural circuit behavior.

**Figure 4:**
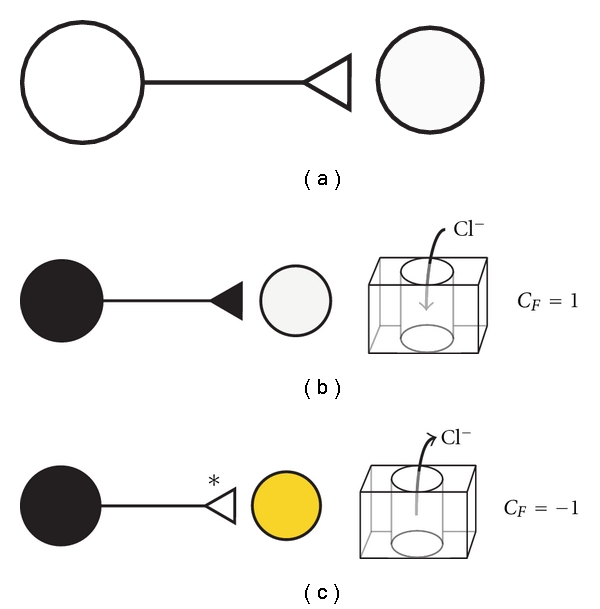
Symbolic representations for normal and aberrant chloride-dependent synapses. Excitatory and inhibitory synapses are denoted based upon the postsynaptic response. The Conversion Factor (*C*_*F*_ = *I*_Cl_*/*I*_Cl_^*o*^) (see (8)) and the chloride flux are shown for chloride-dependent synapses with normal and aberrant (denoted with asterisks) postsynaptic responses. (a) Excitatory neuron with excitatory postsynaptic response. (b) Inhibitory neuron with inhibitory postsynaptic response. (c) Inhibitory neuron with a converted chloride-dependent synapse. The aberrant excitatory response is denoted with an asterisk and an open triangle. Elevated chloride in the postsynaptic cell is denoted with yellow.

 Traditionally, an open triangle is often used to denote an excitatory synapse [25]. An open circle is used to denote the cell body of a pre-synaptic excitatory neuron. Conversely, a dark circle and dark triangle are used to represent the cell body and synaptic terminal of an inhibitory neuron. To denote a chloride-opathy in a postsynaptic CNS neuron, we shade the cell body yellow (Figure 4). To denote synaptic conversion in a chloride-dependent synapse, we utilize an open triangle with an associated asterisk. An asterisk draws attention to a specific synapse that is affected by a chloride-opathy in a neural circuit. In these representations, GABAergic and glycinergic neurons retain dark cell bodies. The terminals of these neurons, however, have an open triangle with an asterisk, denoting an excitatory postsynaptic response. In situations where it is advantageous to express the magnitude of synaptic conversion, the value of the Conversion Factor, *C*_*F*_, can be placed next to the affected synapse.

## 3. Results and Discussion

### 3.1. Synaptic Conversion and Allodynia

**Figure 5:**
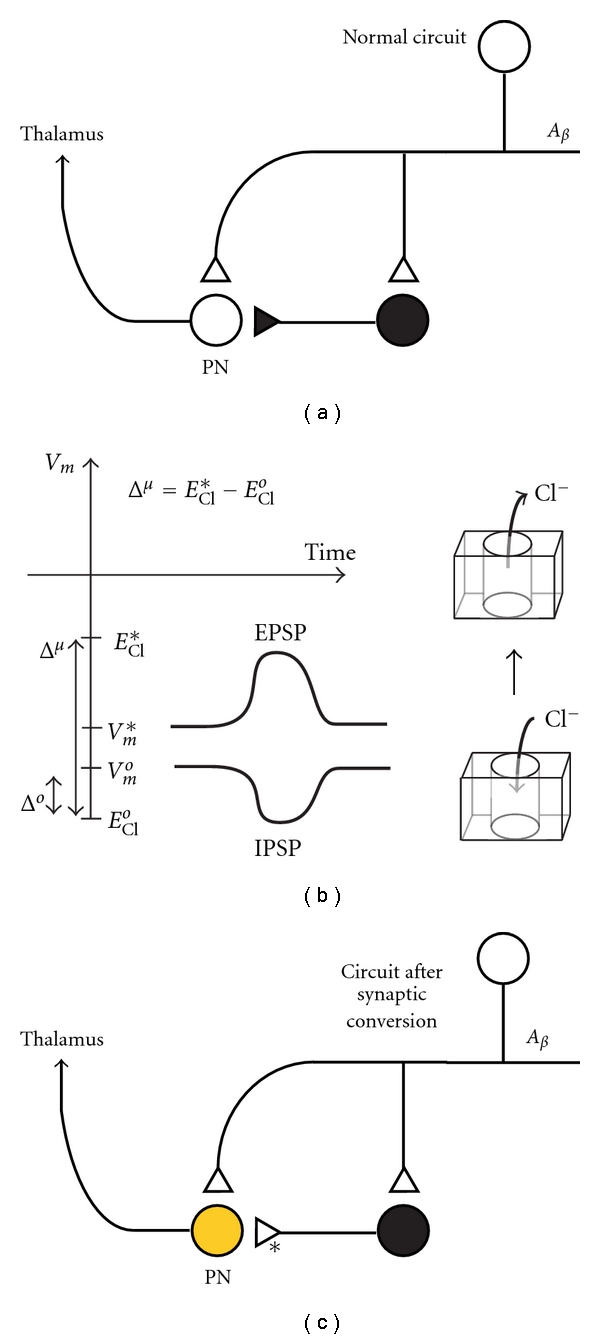
Chloride-opathies and allodynia. Symbolic representation of the concepts and results of Coull et al. [3]. (a) A normal nociceptive neuronal circuit in the mammalian dorsal horn. A myelinated *A*_*β*_ fiber synapses directly onto a lamina I projection neuron (PN), which projects to the thalamus. The *A*_*β*_ fiber also synapses onto a GABAergic inhibitory interneuron, which suppresses PN activity via feedforward inhibition [25]. (b) Electrodynamics of chloride-dependent synapses. After neuronal or vascular injury, microglia in the dorsal horn release BDNF, resulting in a downregulation of KCC2 (Figure 2). This causes an increase in intracellular chloride (Figure 3), and a shift in the normal chloride Nernst potential to a more positive value, *E*_Cl_*. A shift in resting membrane potential may also occur, to a new resting level, *V*_m_*. When *E*_Cl_* > *V*_m_* synaptic conversion occurs in the chloride-dependent synapse, resulting in the generation of excitatory postsynaptic potentials (EPSPs), instead of inhibitory postsynaptic potentials (IPSPs). (c) A symbolic representation of tactile allodynia. When the PN develops high intracellular chloride (yellow), its chloride-dependent synapses undergo synaptic conversion. Activation of the GABAergic interneuron results in the aberrant excitation of the PN, resulting in tactile allodynia. Synaptic conversion has produced a pathology in the *logic* of the nociceptive circuit. Because of the chloride-opathy in the PN, the nociceptive circuit with feedforward inhibition (a) has been converted into a nociceptive circuit that manifests aberrant feedforward facilitation.

 Using the symbols defined in the previous section, we graphically represent a mechanism underlying the onset of allodynia (Figure 5). Primary *A*_*β*_ afferents activate GABAergic interneurons within the upper laminae of the dorsal horn [3]. These GABAergic interneurons synapse onto the same lamina I projection neurons that are innervated by *A*_*β*_ afferents (see circuit model in [25]). With synaptic conversion, GABAergic synapses onto lamina I projection neurons change from being inhibitory synapses to excitatory synapses [3, 6]. 

 The ramifications of the loss of chloride-dependent inhibitory tone in several spinal circuits are profound. Stimulation of *A*_*β*_ fibers normally leads to touch-evoked analgesia. This occurs through the activation of GABAergic interneurons, which normally provide inhibitory tone to lamina I nociceptive projection neurons. After synaptic conversion, an inverted physiological response occurs at this synapse; stimulation of *A*_*β*_ fibers leads to touch-evoked allodynia [3]. 

 Loss of chloride-dependent inhibitory tone can profoundly alter the function of pain circuits. Although the continuity of the nociceptive circuits is intact, the *logic* encoded in the neural circuit can become fundamentally disrupted. For instance, synaptic conversion in lamina I projection neurons [3] leads to a disruption of circuit logic in the dorsal horn by creating feedforward facilitation of nociception, instead of feedforward inhibition—the normal pain gating function of the circuit (Figure 5).

### 3.2. Psycho-Physiologic Pain Amplification

 The experience of neuropathic pain can be highly variable, changing with the mental focus and emotional state of the affected individual [26]. This is because supraspinal and spinal circuits can interact to attenuate, or potentiate, the experience of painful stimuli [26]. In the context of centralized neuropathic pain, how do chloride-opathies at the spinal level influence the transduction of antinociception? This question can potentially be addressed by considering the transduction of pain in patients with Complex Regional Pain Syndrome (CRPS). 

 CRPS is a neuropathic pain disorder characterized by both peripheral and central neuroinflammation, which is often triggered by peripheral nerve injuries [29–31]. Individuals with CRPS frequently have tactile allodynia [32]. A recent postmortem analysis of a patient with long-standing CRPS revealed strong microglial and astroglial activation in the segments of the spinal cord that are ipsilateral and somatotopic to the affected limb [31]. Animal models with neuropathic pain also show both astroglial and microglial activation in spinal segments that are ipsilateral and somatotopic to peripheral nerve injuries [13, 33, 34]. Thus, individuals with CRPS may represent a cohort of neuropathic pain patients, in which both spinal neuroinflammation and spinal chloride-opathies are present.

 Psychoactive pain is prevalent in individuals with Complex Regional Pain Syndrome (CRPS). For a number of individuals with CRPS, the experience of strong emotions [26], and even the ideation of movement of an affected limb, can initiate pain [35]. Thus, the intensity of pain in individuals with CRPS, as well its dependence on mental activities, is far beyond normal experience [26, 36]. 

 Prescott et al. [24] have emphasized that descending GABAergic fibers from supraspinal centers can increase excitability of spinal projection neurons, if the projection neurons have collapsed chloride gradients. A major antinociceptive pathway has become altered into a pro-nociceptive pathway, as a result of microglial activity in the vicinity of the projection neuron. Prescott et al. [24] predict that pain will be generated from normal descending inhibitory GABAergic fibers from the midbrain or brainstem, as they interact with nociceptive projection neurons, which have undergone synaptic conversion of their GABAergic and glycinergic chloride-dependent synapses.

**Figure 6:**
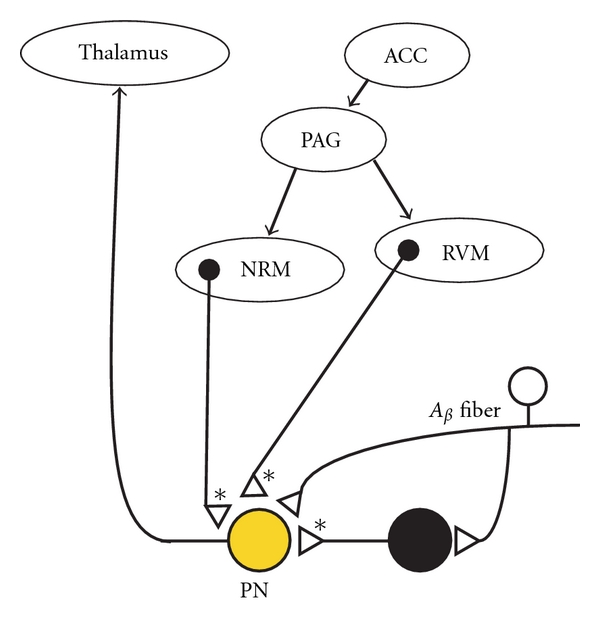
Psychophysiologic pain can potentially arise when descending GABAergic neurons from supraspinal sites drive spinal nociceptive circuits that have been altered by synaptic conversion. The anterior cingulate cortex (ACC) becomes activated during strong emotional states (e.g., anger or fear), as well as during psychological conflict [26]. The ACC also regulates the experience of pain, as well as the anticipation of pain. The ACC drives the periaqueductal grey (PAG) region of the midbrain [27], which in turn drives the nucleus raphe magnus (NRM), as well as the rostral ventrolateral medulla (RVM). GABAergic fibers that descend from the NRM and RVM innervate lamina I projection neurons in the dorsal horn of the spinal cord. When the projection neuron has abnormally high intracellular chloride (i.e., a chloride-opathy), the descending GABAergic drive becomes *pro-nociceptive* [24]. Normally, this drive is antinociceptive. Thus, a major pain coping mechanism, originating at supraspinal levels, is compromised by neuroinflammation and chloride-opathies at the spinal level.

 The anterior cingulate cortex (ACC) is known to regulate distressing aspects of pain, as well as modulate anticipatory responses and mental attention towards pain [26]. In rodents, it has been hypothesized that the ACC produces short-term antinociception by stimulating the PAG [27], which in turn activates inhibitory descending tone to dorsal horn nociceptive neurons [37–39]. Extrapolating this hypothetical pathway to humans, mental activity that activates the ACC would be expected to produce antinociception by driving the PAG [24, 27], which in turn would drive the firing of GABAergic neurons from the nucleus raphe magnus (NRM) and rostral ventrolateral medulla (RVM) (Figure 6) [27, 37, 38]. 

 The above hypothetical transduction pathway would be altered greatly in individuals with spinal neuroinflammation. With synaptic conversion occurring in spinal projection neurons, normal inhibitory tone produced by descending GABAergic neurons from the NRM and RVM would be changed into excitatory tone [24]. An inverted physiological response would be expected to occur as a consequence. Pain would be enhanced by mental states (stress, conflict) that activate the ACC (Figure 6). In contrast, mental distractions of the individual would be expected to attenuate the intensity of neuropathic pain, by reducing the activity of the ACC and the PAG.

 This proposed etiology may help explain observations by Dr. Silas Weir Mitchell [40] during the American Civil War, in soldiers with CRPS. Mitchell described a strong linkage between affective state and pain in certain CRPS patients “…every strong moral emotion made him worse—anger or disappointment expressing themselves cruelly in the aching limb.”

 From a diagnostic perspective, this type of pain could easily be interpreted as psychogenic, because the observed pain is associated with specific types of mental activity. However, the term psychogenic pain has traditionally meant pain originating in the mind [41]. For the conditions diagramed in Figure 6, the term “psycho-physiologic pain” is a more apt descriptor of the pain-generating pathway, which involves supraspinal activation of a disinhibited, spinal nociceptive neuron. From this perspective, the neural circuits with synaptic conversion, shown in Figures 5 and 6, provide a common mechanistic linkage for two types of evoked pain (i.e., tactile allodynia and emotion-linked pain) experienced by CRPS patients.

### 3.3. Transmembrane Bicarbonate Currents and Synaptic Conversion

 When considering pharmacological interventions for chloride-opathies, it is important to emphasize that a number of anions can pass through ligand-gated chloride channels, such as a GABA-A receptor or a glycine receptor [6]. In the nervous system, chloride and bicarbonate ions are the predominant current carriers. Thus, in a GABAergic or glycinergic synapse, it is sometimes important to mathematically delineate how much current is carried by chloride ions versus bicarbonate ions. In normal conditions, chloride fluxes are usually significantly larger than bicarbonate fluxes [6]. However, in situations where chloride gradients have collapsed, that is, *C*_*F*_≅0, the effects of bicarbonate ion fluxes on neuronal excitability become significant [28, 42]. 

 The chloride Nernst potential is an independent variable from the bicarbonate Nernst potential. Thus, the sum of the transmembrane chloride current and transmembrane bicarbonate current can be represented by a transmembrane anion current, defined as:

(11) $$\begin{array}{cc} I_{\text{anion}} & =I_{\text{Cl}}+I_{\text{HC}{\text{O}}_{3}}=G_{\text{Cl}}\left[V_{m}-E_{\text{Cl}}\right]+G_{\text{HC}{\text{O}}_{3}}\left[V_{m}-E_{\text{HC}{\text{O}}_{3}}\right], \end{array}$$

(12) $$\begin{array}{cc} C_{F}^{\text{anion}} & =\frac{I_{\text{Cl}}^{*}+I_{\text{HC}{\text{O}}_{3}}^{*}}{I_{\text{Cl}}^{o}+I_{\text{HC}{\text{O}}_{3}}^{o}}\\ & =\frac{G_{\text{Cl}}\left[\Delta_{\text{Cl}}^{o}+\Delta^{V}-\Delta_{\text{Cl}}^{\mu }\right]+\left[\Delta_{\text{HC}{\text{O}}_{3}}^{o}+\Delta^{V}-\Delta_{\text{HC}{\text{O}}_{3}}^{\mu }\right]}{G_{\text{Cl}}\cdot \Delta_{\text{Cl}}^{o}+G_{\text{HC}{\text{O}}_{3}}\cdot \Delta_{\text{HC}{\text{O}}_{3}}^{o}}. \end{array}$$

For clarity, the driving potentials for Cl and HCO_3_ ions are specifically identified with subscripts*. *Changes in the chloride and bicarbonate chemical potentials from norm-averaged values are represented by:

(13) $$\begin{array}{c} \Delta_{\text{Cl}}^{\mu }=E_{\text{Cl}}^{*}-E_{\text{Cl}}^{o},\\ \Delta_{\text{HC}{\text{O}}_{3}}^{\mu }\;=E_{\text{HC}{\text{O}}_{3}}^{*}-E_{\text{HC}{\text{O}}_{3}}^{o}. \end{array}$$

In GABAergic and glycinergic synapses where the chloride driving potential (i.e., Δ_Cl_) has a value close to zero, the anion channel Conversion Factor reduces to

(14) $$C_{F}^{\text{anion}}=\frac{I_{\text{Cl}}^{*}+I_{\text{HC}{\text{O}}_{3}}^{*}}{I_{\text{Cl}}^{o}+I_{\text{HC}{\text{O}}_{3}}^{o}}=\frac{G_{\text{HC}{\text{O}}_{3}}\left[\Delta_{\text{HC}{\text{O}}_{3}}^{o}+\Delta^{V}-\Delta_{\text{HC}{\text{O}}_{3}}^{\mu }\right]}{G_{\text{Cl}}\cdot \Delta_{\text{Cl}}^{o}+G_{\text{HC}{\text{O}}_{3}}\cdot \Delta_{\text{HC}{\text{O}}_{3}}^{o}}.$$

In cases where Δ_Cl_≅0 and Δ^*V*^ = 0, GABAergic synapses have a Conversion Factor approximated by:

(15) $$C_{F}^{\text{anion}}=\frac{I_{\text{HC}{\text{O}}_{3}}^{*}}{I_{\text{Cl}}^{o}+I_{\text{HC}{\text{O}}_{3}}^{o}}=\frac{G_{\text{HC}{\text{O}}_{3}}\left[\Delta_{\text{HC}{\text{O}}_{3}}^{o}-\Delta_{\text{HC}{\text{O}}_{3}}^{\mu }\right]}{G_{\text{Cl}}\cdot \Delta_{\text{Cl}}^{o}+G_{\text{HC}{\text{O}}_{3}}\cdot \Delta_{\text{HC}{\text{O}}_{3}}^{o}}.$$

Note that the bicarbonate current remains referenced to the chloride driving potential for normal conditions, as well as the bicarbonate driving potential for normal conditions. Use of fixed reference points is appropriate for a mathematical scaling factor. Deviations in bicarbonate driving potential from norm-averaged conditions are accounted for by the mathematical term, Δ_HCO_3__^*μ*^. 

 In spinal nociceptive neurons, in which chloride gradients have collapsed (i.e., Δ_Cl_≅0), outward flux of HCO_3_ can produce significant depolarizing currents, leading to the generation of action potentials and pain. The magnitude of these depolarized bicarbonate currents can be approximated with the following equation:

(16) $$I_{\text{anion}}^{*}=C_{F}^{\text{anion}}\left(I_{\text{Cl}}^{o}+I_{\text{HC}{\text{O}}_{3}}^{o}\right)=C_{F}^{\text{anion}}G_{\text{HC}{\text{O}}_{3}}\cdot \Delta_{\text{HC}{\text{O}}_{3}}^{o}.$$

Modifications of the bicarbonate gradient will change the magnitude of the Conversion Factor shown in (15).

### 3.4. Pharmacological Treatments for Chloride-opathies

 Clinical management of chloride-opathies differs substantially from the clinical management of channelopathies [6]. This is because neuropharmaceuticals have traditionally been designed to alter neuronal excitability through changes in ionic conductance. Pharmacological agents can compensate for the loss of chloride-dependent inhibitory tone by reducing overall neuronal excitability. However, they do not alter the collapsed chloride gradient, the underlying cause of the chloride-opathy.

 Recently, anion transport inhibitors have been repurposed for the treatment of neuroexcitability disorders linked to aberrant chloride homeostasis [42]. For example, bumetanide has been used to treat epilepsy in neonate infants [19]. By inhibiting inward chloride cotransport via NKCC1 (Figure 2), bumetanide partially restores the transmembrane chloride gradient across postsynaptic membranes [6]. This occurs even though the activity of KCC2 remains deficient, and may explain the clinical benefit of bumetanide in cases of epilepsy where KCC2 transcription in cortical neurons, or hippocampal neurons, is suspected to be abnormally low [19].

 An entirely new approach to treat chloride-opathies in the CNS is to compensate for aberrant chloride-dependent inhibitory tone by altering bicarbonate fluxes through GABA-A and glycine channels [42]. First, benzodiazepines are used to open GABA-A channels. This treatment maximizes anion conductance through the postsynaptic membrane. A carbonic anhydrase inhibitor (acetazolamide) is also applied to suppress transmembrane bicarbonate fluxes by reducing intracellular bicarbonate formation [42]. The outcome of applying the benzodiazepine, as well as the carbonic anhydrase inhibitor, is a synergistic, antiallodynic effect. Decreased bicarbonate flux reduces excitatory drive through extrasynaptic GABA-A receptors. At the same time, an augmented shunting inhibition through postsynaptic GABA-A receptors is preserved [42]. The net result is a suppression of pain generated at the spinal level. 

 For this particular situation, the generalized Conversion Factor, *C*_*F*_^anion^ (see (14)), can be used to represent the altered postsynaptic response of GABAergic and glycinergic synapses in cells treated with carbonic anhydrase inhibitors. Assuming transmembrane chloride gradients are completely collapsed, (i.e., Δ_Cl_ = 0), transmembrane anion current is represented by (16). The net current through the postsynaptic membrane is depolarizing because of the outward bicarbonate flux. 

**Figure 7:**
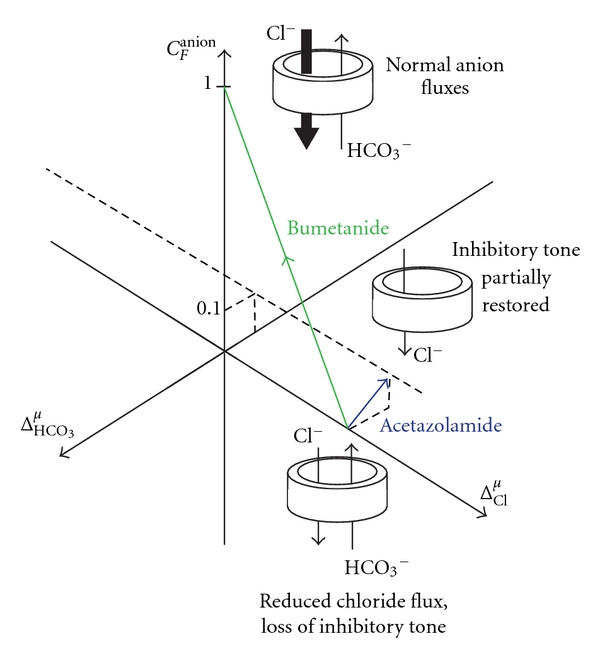
Pharmacological management of chloride-opathies. *C*_*F*_^anion^ dependence on Cl and HCO_3_ driving potentials (see (12)). In the above hypothetical case, the *I*_Cl_^*o*^/*I*_HCO_3__^*o*^ratio for normal anion currents through ligand-gated chloride channel (a GABA-A or a glycine receptor) is assumed to be 10 : 1. No change in resting membrane potential occurs (i.e., Δ^*V*^ = 0) in this example, as anion driving potentials deviate from their norm-averaged values (Δ_Cl_^*μ*^ and Δ_HCO_3__^*μ*^). At *C*_*F*_^anion^ = 0, the inward flux of chloride is counterbalanced by an equal and opposite flux of HCO_3_. Chloride-dependent inhibitory tone in the affected synapse has been lost. Bumetanide, an inhibitor of NKCC1 (sodium-potassium-chloride cotransporter 1), reduces the transport of chloride into the postsynaptic neuron (Figure 2). With a reduction of intracellular chloride, *C*_*F*_^anion^ moves back towards a normal value of 1. A novel approach to restoring a positive value of *C*_*F*_^anion^ is to reduce intracellular bicarbonate (HCO_3_) concentration with the carbonic anhydrase inhibitor, acetazolamide [28]. This occurs while the collapsed chloride driving potential, Δ_Cl_^*μ*^, remains unchanged. Bumetanide and acetazolamide have antiallodynic actions in animal models of neuropathic pain [6].

 To consider the action of potential pain therapeutics, it is useful to consider the special case of when inward chloride and outward bicarbonate currents exactly counteract each other (i.e., *C*_*F*_^anion^ = 0). The bicarbonate gradient across the postsynaptic cell membrane can be collapsed by the application of a carbonic anhydrase inhibitor, acetazolamide, which inhibits bicarbonate formation on both sides of the cell membrane. As a result, the generalized anion Conversion Factor can become nonzero (Figure 7). This restoration of inhibitory tone is believed to correspond with the antinociceptive action of acetazolamide [42]. 

Figure 7 illustrates the interplay between chloride and bicarbonate driving potentials. In addition, the therapeutic actions of bumetanide and acetazolamide are qualitatively portrayed. Bumetanide moves the anion Conversion Factor towards a more positive value, by partially restoring a normal transmembrane chloride gradient. Acetazolamide moves the anion Conversion Factor toward a more positive value, by reducing an outward (depolarizing) bicarbonate current. When a residual inward chloride current is counterbalanced by an outward bicarbonate current, the generalized anion Conversion Factor is zero. An increase in the magnitude of the anion Conversion Factor above zero indicates that the synapse has become hyperpolarizing, thereby restoring some chloride-dependent inhibitory tone to the postsynaptic neuron.

### 3.5. Chloride-opathies and Neuroinflammatory Disorders

 Widespread evidence now indicates that a large range of pain, neurodegenerative, motor, and affective disorders involve neuroinflammatory processes mediated, in part, by activated microglia [13, 43–45]. Activated microglia are associated with Alzheimer's Disease, Parkinson's Disease, Huntington's Disease, multiple sclerosis, amyotrophic lateral sclerosis (ALS), spasmodic dysphonia, and chronic neuropathic pain disorders, including painful diabetic neuropathy [43–49]. Activated microglia have also been found in the brains of autistic children [50], as well as individuals with schizophrenia [51]. 

 In each of the above disorders, it is important to consider how microglial-mediated chloride-opathies could produce functional changes in neural circuits. Loss of chloride-dependent inhibitory tone in specific spinal and/or supraspinal sites could produce distinctive nociceptive, autonomic, proprioceptive, and/or motor morbidities. For example, downregulation of KCC2 in human cortical or hippocampal neurons can lead to epileptic seizures [52, 53], whereas downregulation of KCC2 in spinal motor neurons in an ALS rodent model leads to muscle weakness [54]. Aberrant chloride cotransport is suspected to be an etiological factor in schizophrenia [55]. Whether aberrant chloride homeostasis arises from activated microglia [55], which are present in the hippocampi of schizophrenic patients [51], remains to be determined. 

 Downregulation of KCC2, and upregulation of NKCC1, in the trigeminal subnucleus caudalis in rodents correlates with the establishment of oral cavity neuropathic pain following dental pulp inflammation, induced by lipopolysaccharide [56]. KCC2 downregulation in these trigeminal nociceptive neurons has been hypothesized to be mediated by a BDNF-TrkB pathway, based upon the antinociceptive actions of K252a, a TrkB antagonist. The presumed pain transduction pathway in the brainstem [56] is thought to parallel the well-characterized BDNF-TrkB-KCC2 pathway that mediates microglial-mediated pain in the dorsal horn of the spinal cord [3, 19].

 From PET (positron emission tomography) imaging studies, it is known that microglial activation can spread from site-to-site through the neuraxis, presumably by chemical signals conveyed by axonal transport [57]. This type of remote neuroimmune activation [58] appears to be involved in the establishment of neuroinflammation in the thalamus, following peripheral nerve injury [57]. In a PET imaging study of chronic pain patients, persistent microglial activation was found in the thalamus, up to two decades after the healing of a peripheral nerve injury [58]. In rodents, sensitization of the thalamus can lead to widespread allodynia throughout the body [59]. In addition, microglial cell and astroglial cell activation in the rostroventromedial medulla (RVM) has been correlated with hyperalgesia in a rodent model of peripheral inflammation [60]. Thus, the generation of neuropathic pain can become multi-focal, involving glial cell activation in both spinal and supraspinal sites. Although glia activation in supraspinal centers has been correlated with centralized neuropathic pain [57, 59, 60], chloride-opathies in supraspinal sites have not yet been causally connected with neuropathic pain. 

 At the cellular level, mathematical models of chloride-opathies could be developed to describe: (a) influx of chloride through chloride channels, (b) rate constants for different chloride cotransporters, (c) diffusive transport of chloride and bicarbonate within defined cell volumes, and (d) the biochemical and genetic regulation of anion transporters. Mathematical models that have already been published account for shifts in intracellular chloride concentration resulting from the activation of GABA-A chloride channels [61–63]. 

 To date, the analysis of chloride-opathies in the CNS has focused primarily on the upregulation and downregulation of two chloride cotransporters, NKCC1, and KCC2. There are, however, a number of other chloride cotransporters in the CNS that contribute to overall intracellular chloride homeostasis [64]. A comprehensive quantitative mathematical model of chloride-opathies, and their effect on chloride-dependent synaptic tone, will need to account for these chloride cotransporters as well. 

 From imaging studies, is it known that transmembrane chloride gradients are dynamic, and regionally different across the dimensions of individual neurons [65]. Sexual dimorphism in the regulation and expression of chloride cotransporters [66] might contribute to phenotypic differences in the frequency of neuropathic pain between females and males. The promoter of the KCC2b isoform, for instance, is regulated by upstream stimulating factor (USF) proteins, which interact with many different signaling cascades [65]. Genetic polymorphisms, tissue-specific isoform expression patterns, as well as mutations of chloride cotransporters are also known [67–70]. All of the above factors could contribute to normal and pathological variations of the transmembrane anion gradient across postsynaptic membranes, which is now recognized as a key integrative factor in the transduction of pain.

## 4. Conclusions

 In this paper, we have developed scaling factors to describe the altered electrical behavior of chloride-dependent synapses in conditions where chloride or bicarbonate ion gradients are aberrant. These conversion factors predict the behavior of the synapse in response to a change in chloride and/or bicarbonate electrochemical driving potentials. These conversion factors can be used to describe synaptic behavior, with or without, changes in the electrical driving potential, Δ^*V*^, for these anions. 

 We have mathematically separated key system variables for anion synaptic currents in our derivation of the generalized Conversion Factor. This mathematical approach allows the contributions of chloride gradients (i.e., Δ_Cl_^*μ*^) and bicarbonate gradients (i.e., Δ_HCO_3__^*μ*^) to be independently discerned from changes in resting membrane potential (i.e., Δ^*V*^). In this regard, the mathematical and symbolic representations of synaptic conversion, which we have developed in this paper, help parameterize and illustrate the critical roles that anion electrochemical driving potentials play in the transduction of pain. 

 Because transmembrane chloride gradients in nociceptive projection neurons are linked to the actions of neighboring activated microglia, the generalized Conversion Factor, *C*_*F*_^anion^, can be viewed as a system parameter that describes the action of their GABAergic and glycinergic *tetrapartite synapses*, that is, anion-dependent neuronal synapses whose functions are modified by neighboring glia. From the standpoint of synaptic tone,  *C*_*F*_^anion^ is a measure of a postsynaptic neuron's response to GABAergic and glycinergic input. Functional roles of chloride-opathies (i.e., pathological chloride driving potentials) in the genesis of neuropathic pain can be defined, and quantified, using this parameter. Symbolic representations of synaptic conversion (Figure 4) can be used to illustrate how aberrant anion homeostasis disrupts the integrative logic of spinal nociceptive circuits (Figures 5 and 6).
